# Reconstructing Personal Stories in Virtual Reality as a Mechanism to Recover the Self

**DOI:** 10.3390/ijerph17010026

**Published:** 2019-12-18

**Authors:** Iva Georgieva, Georgi V. Georgiev

**Affiliations:** 1 Institute for Advanced Study, 9010 Varna, Bulgaria; ivavgeorgieva@gmail.com; 2 Department of History and Philosophy of Science, The University of Tokyo, Tokyo 153-8902, Japan; 3 Center for Ubiquitous Computing, University of Oulu, 90014 Oulu, Finland

**Keywords:** philosophy, virtual reality, narrative self, personal story, trauma, narrator, protagonist, author, recovery, growth

## Abstract

Advances in virtual reality present opportunities to relive experiences in an immersive medium that can change the way we perceive our life stories, potentially shaping our realities for the better. This paper studies the role of virtual reality as a tool for the creation of stories with the concept of the self as a narrator and the life of the self as a storyline. The basis of the study is the philosophical notion of the self-narrative as an explanatory story of the events in one’s life that constitutes the notion of one’s self. This application is suitable for cases when individuals need to recreate their self, such as during recovery after traumatic events. The analysis of the effects of virtual reality shows that it enables a person to engage in a process of deeper self-observation to understand and explain adverse events and to give meaning to these events to form a new story, which can complement the therapeutic outcomes of exposure treatments. This study proposes concrete examples of immersive scenarios used to reconstruct personal stories. Several possible levels of experience are proposed to suggest that recovery can be achieved through the gradual retelling of the self-narrative, addressing all of the underlying narratives. Considering the ethical challenges that might arise, this paper explores the ways in which immersion in virtual reality can benefit a person’s view toward life as a story and his or her self as its author, comparing this idea with previous research on the application of virtual reality for trauma treatment. The analysis also emphasizes the perception of narrative authorship in virtual reality as an essential method for recovering the self-narrative and improving a patient’s mental health during self-actualization.

## 1. Introduction

Since the emergence of virtual spaces, studies of their effects on self-identity have suggested many theoretical frameworks [1,2,3,4,5,6,7] to understand how these spaces can shape our experiences [8,9,10] and self-representations [11,12,13,14]. As one of the most sophisticated mediums for virtual experiences, virtual reality (VR) [15] has been analyzed in studies that see multiple effects on the self and the views people hold toward reality [16,17,18].

This research connects the concepts from these studies with ethical and philosophical considerations of the self-narrative [19,20,21] to further explain the effects of VR on the self during the recovery of a narrative developed over a lifetime, especially after traumatic events. The paper specifically emphasizes the need for scenarios with more extensive theoretical background and refined methodology to use VR for the treatment of mental health issues as greater research into its applications in digital health is required [22]. This is a theoretical study based on the philosophical idea of the narrative self, which constitutes the human self-identity and can be influenced through experiences in virtual environments. As this is a focus on the philosophical account of trauma, it complements the psychological clinical perspectives on the subject. The main goal of this paper is to suggest improvement methods for the treatment of post-traumatic stress disorder (PTSD) through the use of narrative VR by emphasizing the importance of taking into account the narrative self in therapy and the opportunity that VR offers in this direction.

### 1.1. Background

The use of VR for entertainment is widely recognized and well developed [23,24] as VR is deeply based on the principles of narrative and storytelling [25]. In addition to its entertainment purposes, VR has more serious applications [26] such as training, treatment, and relaxation. However, the healthcare applications of VR have mainly focused on the recreation of past events and existing scenarios for exposure with habituation purposes [27,28,29], without addressing the main challenge that is present in the fact that trauma is a deeply personal experience shaped by the story one has about their life as well as a previous history of traumatic events that creates a traumatized self-perception and view upon reality. Specifically, this research supports the idea that the theoretical account of the philosophy of narrative self-identity can better serve the clinical application of VR for the treatment of traumatic disorders with the consideration of the personal context surrounding the traumatic events and the story each patient has about their life. Clinical applications of VR to treat mental health conditions such as PTSD suggest that VR has significant potential to address and alleviate such issues [30] as well as a full-range of mental health issues [31]. Results obtained from such studies show that the clinical application of VR offers the potential to create environments that allow for the control of complex, immersive stimulus presentations allowing interaction, behavioral tracking, user response, and performance recording, together with therapeutic intervention from the clinician [32].

It is necessary to consider why VR can offer alternative treatment solutions with its ability to offer different viewpoints of problems not otherwise visible. Through VR, one can visualize stories in new ways through the idea that narratives shape reality and people’s perception of events.

The narrative potential of VR for creating stories similar to those experienced and adding meaning and context to them is the basis of this paper’s conceptual framework [33]. We consider the use of VR in therapy as an advanced narrative medium to further specify the treatment content and address each personal case in detail, as this issue is becoming increasingly relevant to the field of cyberpsychology [34,35]. The paper aims to present preliminary conclusions regarding how therapies can be constructed based on the narrative self and to outline proposals for the development of immersive scenarios to reconstruct these self-narratives in gradual steps to uncover all the narratives that may prevent a patient’s recovery. As research has shown, VR exposure therapy has gained its merits through the possibility of offering gradual exposure as required in cases of acute PTSD [36].

### 1.2. Motivation of the Research

The central concept of this paper is that VR can be used to create more meaningful narratives that can help the patient reconstruct life events, specifically traumatic ones, and integrate new explanations, thereby complementing the effects of exposure treatment. It is intended to be used to discover new opportunities for the clinical application of VR, which generally involves emotional processing, habituation, cognitive restructuring for new responses to dangerous stimuli, and integration of non-traumatized information into the belief structure [37]. In this sense, it is possible to consider the treatment as preventing negative experiences from establishing one-way false beliefs about reality such as “the world is a dangerous place” [38]. Moreover, the experiences that one can gain through exposure in VR can show that there are many options for the active creation of reality depending on personal choice, interpretation of events, and view upon the world. Even though events cannot be changed, one can experience similar in association but different in tone events to overwrite and overcome ones that they cannot change, so that they feel able to own different narratives about themselves. This could happen by engaging in VR narratives that connect with cathartic experiences after the initial exposure and cognitive restructuring work to be done in cybertherapy.

For this, the main theoretical idea is that humans constitute their selves using narratives (i.e., a person creates their identity by forming an autobiographical interpretational story about the events in their life). Such reconstruction would help to recover the unity of the self and improve the overall health of the person behind that self [39]. A therapeutic strategy of VR in its storytelling essence can complement current VR-based approaches; through the use of narrative VR, limitations can be overcome and notable treatment results could be achieved by employing the philosophical account of the self-narrative as the basis of a storytelling therapy in VR.

## 2. Application of Immersive Technologies in Healthcare

### 2.1. Recreative Immersion

The creation of various medical environments with healthcare impacts and personalized experiences can be aided by emergent immersive technologies such as VR, augmented reality (AR), mixed reality (MR), or cross reality (XR), which have become popular as the basis of recreational games [40,41,42,43,44,45,46]. While AR, MR, and XR, in general, can provide training, education, relaxation, or entertainment, VR is the medium that presents the opportunity to most efficiently recreate events that are part of personal narratives, to deeply engage the immersed person with the highest sense of presence [47,48,49,50,51], and most efficiently simulate the personal narrative of the self [52]. VR offers not only the opportunity to elicit a strong response to stimuli, but also provides an as if quality, meaning that it can be compared to the real impact of an experience [53]. The future of VR in healthcare requires deeper research for the whole range of effects it can have on the human self, and its narrative understanding of reality to be realized [54,55].

VR is a powerful, flexible, and multifaceted tool that can be aided by artificial intelligence (AI) to design various scenarios with plausible and rich stories that flexibly answer the changing needs of those immersed in the medium [56,57]. The combination of VR and AI can create a well-developed flow of narrative stories that can be used for specific cases such as PTSD, a condition that can be triggered when the patient experiences circumstances similar to the traumatic event. The design of the immersion, however, must be adapted to the personalized story of each individual [58,59]. Such future applications of AI in VR can contribute to faster treatment results and broader effects on the overall health and wellbeing of the general population [60,61,62].

### 2.2. Virtual Reality as An Ultimate Narrative Medium

This paper addresses the possibility of using VR as a medium to re-create one’s life story and re-experience one’s self as the author of this story [33]. On the basis that self-narratives constitute personal identity, or one’s self, one can achieve a re-creational effect on the self in a VR environment, especially when faced with challenging situations. This is possible because VR shares the potential of the narrative mediums for storytelling such as books or movies, but offers an advanced environment for reliving past events. VR can provoke such emotions as compassion, empathy, and a sense of agency or ownership, similar to how people sympathize and relate with characters in books or movies that help them identify the outcomes of stories *as if* they had achieved them themselves. Due to this experience, the audience achieves catharsis or personal growth as a conclusion to these stories.

Moreover, the effects of VR experiences could help one regain authorship of one’s life story through the immersion and construction of a healthier narrative meaning. Specifically, this research proposes methods for the development of narratives to recreate the self as well as different models of stories to build in VR, based on the same concept. Consequently, the investigation addresses ethical challenges and limitations faced in specific traumatic cases found within the different types of PTSD.

The focus on the many possible uses of VR as a treatment medium is expanding as various studies try to identify how the medium can be adapted to expand beyond mere exposure treatment and achieve significant cognitive restructuring and treatment results [32,63]. However, although many studies have emphasized the personalization of the treatment design [64,65], they do not necessarily employ conceptual frameworks regarding the diverse types of trauma in the case of disorders such as PTSD; in particular, they do not consider the narrative nature of the human self that plays a significant role in the interpretation of life events and hence forms a specific perception of reality and related health conditions.

Compared to other studies, this research emphasizes the role of the medium in experiencing alternatives of the self, obtaining resolutions, and adding meaning in the context of one’s life story through enhanced design of the VR scenarios. With this, its contribution is in the theoretical proposal for a better understanding of the meaning behind the story and achieving authorship in the narrative of the self through the VR experience. The ideas proposed here are distinct from those of existing studies on the subject and their implications [66,67,68,69,70,71] that might rely on customization with the narrative concept, but do not fully consider the potential of the storytelling role of VR in matching application design and purpose in the context of narrative therapy.

## 3. Self-Narrative and Its Application in Therapy

To explain the benefits of employing a philosophical account on the narrative self, it is necessary to emphasize its claim that “human brains are narrative-generating machines and selves are the protagonists of the narratives they generate” [21]. Hypothetically, this indicates that it is possible to consider psychological trauma as a break [33,72] in the narrative of the self, as supported throughout this research, and design the treatment narrative accordingly. For example, PTSD patients often have difficulty living in the present [73,74,75]. In such cases, more robust therapeutic results require an understanding of how to interpret, explain, accept, and intertwine the traumatic event into an overarching and consistent narrative of human life, acknowledging the many storylines and backgrounds that form the self and its life story [38,76,77,78]. In other words, to achieve therapeutic recovery, it is necessary to consider how to use the narrator’s ability to form a new narrative and to explain the traumatic event as a meaningful part of their life story [79], which can be further aided by utilizing the storytelling characteristics of VR.

A previous study on storytelling in VR proposed that “[a] story is a sequence of events or scenarios that demonstrate characters trying to resolve conflict by accomplishing goals” [25]. A traumatic event could never be considered as a desirable event in a human life; however, an explanation and self-efficacy can be discovered amidst hardship [80,81,82], along with other beneficial results, for example

- reclaimed authorship;
- improved resilience; and
- personal growth.

PTSD can be a devastating health condition. However, post traumatic growth [83,84,85], the desire to change one’s perspective and to see gain within loss [86], the transformational power of focusing on positive interpretations [87], and different explanations for the past that can lead to growth rather than decay are some of the tools that can be used to fight and overcome the effects trauma has on one’s life. This connection between the narrative and post traumatic growth highlights the natural human ability to reassess a personal narrative (i.e., to achieve cognitive restructuring) and find resolutions in seemingly unresolvable outcomes in different processes and contexts of recreating the self [88,89,90,91]. The way trauma affects each personal narrative has been the subject of much research [92]; in this study, we would like to consider these findings and put them into the context of the personal growth people learn and experience through stories about life and the meanings they attach to these stories [93] while utilizing the power of VR in visualizing and altering one’s storyline to recover the self [38].

The way in which people perceive any story as a part of their life and then narrate the event for future generations has always been a method for reassessing and processing life’s adversities to create resilience rather than distress [94] and achieve growth through healing [95,96,97]. Traditional storytelling sees the hero’s journey as a difficult cycle of transformation that helps them to overcome difficulties and continue their life in a better way. The striving of the human spirit to achieve this development is not itself a new story; however, we now have the tools to recreate such stories in a convincing and impactful way through VR.

This paper will describe both the benefits and the limitations of adopting such a narrative therapy and storytelling methods via comprehensive analysis of the novelty of the proposal in relation to other studies [98,99,100] while considering possible obstacles to the desired therapeutic effects from the application of such treatment. This, however, cannot be an extensive literature review on the subject as the paper proposes a very different conceptual framework based on the philosophical concept of the narrative self, which is theoretically difficult to compare to those usually discussed in clinical evidence literature.

## 4. VR as a Medium for Understanding the Self-Narrative

**Figure 1:**
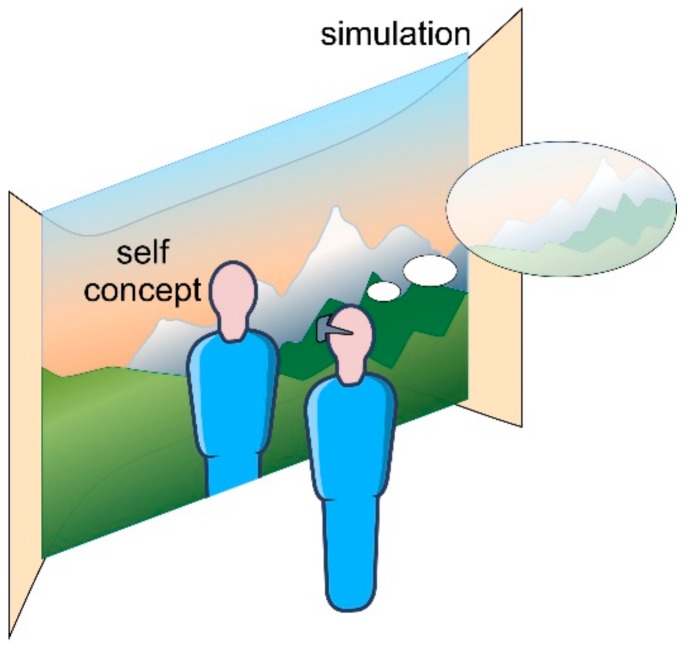
Virtual Reality seen as a “simulation.” Virtual Reality is considered as a simulation of the external reality; however, even as such, it can represent things that are not seen and understood in reality in a distanced way such as the way one appears in reality or how he or she forms his or her image of the world. Even though Virtual Reality is first perceived as “virtual”, exactly in it one can see that their view toward the world is somewhat blurred or fading because of the trauma. As a result, from such a realization, one can see clearly that trauma impacts self-image and world view, and understands objectively that their world is not as broken as they imagine and that it could be fixed.

The self-narrative can be understood through exposure in VR, where people can easily play with the creation of new features of their virtual self [15], as they can believe it is only a simulation, a mirror, or a copy of their real life. The combination of the realistic or mirror-like and the fantastic effect VR has can be used for a wide range of purposes not limited to treatment, and showing diverse influences on self-image and reality concepts [101,102]. However, because exposure in VR offers a new perspective [103] through novel utilization of the storytelling medium [104], a person finds themselves immersed and starts changing their self-concepts more quickly and easily than in reality (see Figure 1).

In the virtual space, people can check to see if they “feel OK” or see if something is wrong and needs to be fixed; this is similar to checking one’s facial expression in a mirror. One application of this process is in correcting body images in VR exposure for obesity therapy [105,106,107,108].

VR can be designed to show either an observer’s point of view or a first-person perspective; therefore, it is possible to identify with the narrative of the self and through exposure to certain stimuli in the virtual world to confirm a person’s reaction to these stimuli and their overall psychological condition [109,110] (as seen in Figure 1, which shows an example where the person might be “seeing” duller colors due to a depressed mental condition). 

**Figure 2:**
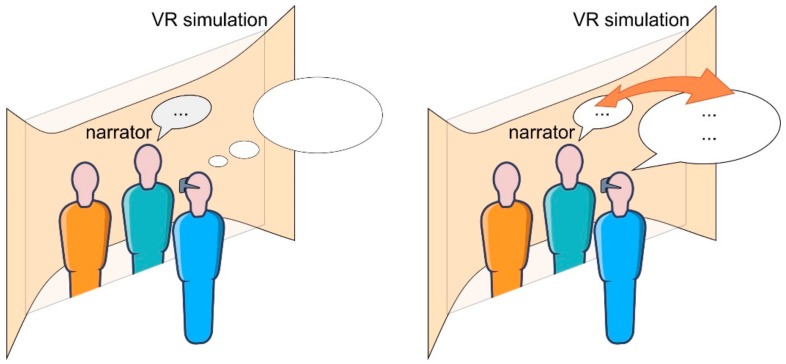
Narrator and protagonist in Virtual Reality. In cases when a person needs guidance in the Virtual Reality exposure, a therapist might appear in the role of a narrator from a typical storytelling setting and perform exchanges with the immersed person to guide and affect them during the experience. Instead of just having the narrator lead the protagonist in the Virtual Reality simulation (left), the narrator (the therapist) might converse with the person exposed to the Virtual Reality simulation in real time (right) to assess changes in perception and cognitive models that become evident during the experience to achieve restructuring of unhealthy thinking patterns.

VR exposure studies rely on re-experiencing stimuli similar or identical to the traumatic event to bring about habituation or resolution of negative emotions associated with the trauma through gradual, controlled, and increasingly sophisticated scenarios [111,112]. However, if VR is used to further improve a person’s health under the consideration of the self as a narrative unity, it is possible to dive further into the experience and start acting upon scenarios that differ from the real event by relying on the principles of storytelling and the idea that through narrative experiences, growth can be achieved. This treatment should be managed by a therapist who can guide the storyline to obtain advanced therapeutic results from the virtual experience, or to intervene when a different mindset is necessary to improve cognitive imbalances (i.e., to achieve cognitive restructuring) [38,113,114,115] (Figure 2).

Therefore, other “reality” options such as AR and MR enhance reality perception, while VR proposes an alternative, but sufficiently immersive reality that can bring about transformation for the self [16,38]. While in AR and MR it can be difficult to obtain full immersion, the realism of VR can make it difficult to distinguish the impression it has from the sensations of a real experience. It is this effect that allows VR to be used to intentionally change one’s life story and its explanatory mechanisms [116]. Previous studies have described the so-called Proteus Effect [3], which is related to the process of transferring experience from the virtual to the real [15,33]. This effect is sought in the treatment proposal here, but also in terms of differing from the previous transformational experiences.

### Seeing Psychological Trauma as A Break in the Narrative and Its Consequences

To further illustrate the narrative self-account, it is important to note that “[t]he narrative of a life can be one with multiple subplots, digressions, and deviations from the main narrative stream; it need not be linear” ([16], p. 342). Therefore, the unity of human life [117] can be seen as broken [33,72] and the coherence of the self as disturbed [118] in conditions resulting from traumatic events. However, in the context of recovery therapy [119], this should not be seen as an irresolvable situation, and it is necessary to create another narrative or fiction that “works” [19] to create meaning out of the traumatic event and to include it with consistency in one’s life story.

Creating meaning is a much more difficult task if moral conflict and ethical challenges are present and if technology itself poses ethical concerns [103,120]. A more or less appropriate reaction to unfortunate events and the ability to help or save others resonates with a person’s self-evaluation or feeling of self-worth and might create further negative effects from the traumatic experience, especially if not realized [121,122,123]. It may be possible to resolve these ethical issues in VR by adapting it with the help of AI to the different types of trauma to alleviate complex cases where trauma is hidden at a deeper level. Simulating a reflection on the values and ethics of a person’s core beliefs [124] can help them construct a more robust and consistent personal narrative to overcome problems and live within a healthier mindset.

This could happen more efficiently in VR than in other (and especially weak storytelling) environments with exposure purposes such as AR or MR. However, the highly immersive feature of VR should be carefully considered, as it might provoke strong reactions, addiction, and even the desire to escape reality [15,33], possibly sharpening moral conflicts [125] that would hinder the healing process. Further ethical and technological problems will be discussed later in Section 7 regarding the challenges and limitations to better grasp the conceptual differences between them.

## 5. Storytelling Techniques and Therapy

This paper explores the philosophical, ethical, and technological circumstances of the notion that retelling a story in VR can have a beneficial impact on the user and experiences it as the author of the story. The study presents a conceptual framework and proposes possible scenarios to address the transformation of the personal narrative through storytelling exposure in VR. To situate this proposal in the context of other therapeutic designs, it is necessary to mention the cognitive behavioral therapies and narrative therapies with exposure in VR and their connection with the idea of the self-narrative as constituting the entity of healthy individuals. As these studies are few in number, similar approaches will be discussed to better describe the general idea of this paper.

### 5.1. Previous Works

The prospects of VR as a storytelling medium for varying purposes increase dramatically each year as the ability to guide the experience during immersion increases the effect of the presence that the medium has on the person experiencing it [126,127]. When this is applied to the treatment of disorders such as PTSD and similar mental health issues, it offers many benefits to the therapy such as the dissolution of barriers such as stigma or cost [128,129]. The serious games application of VR offers various scenarios and generates scenery according to the advancing exposure [130]. Specific fields of treatment such as within the military have for a long time found a resolution of acute problems through therapy in VR; there have, however, been criticisms of the technology, for example, fears it turns its users into “machines” [131,132,133].

To summarize, the research focusing on the use of VR for treatment in relation to this paper can be defined in the following topics, which are often combined within the therapeutic setting:

- Exposure therapy;
- Cognitive restructuring/cognitive behavioral therapy (CBT); and
- Narrative therapy.

An important point supported by this research is that VR enables customization of the environment for the specific needs of the treatment, which is essential as PTSD itself manifests in many different ways [134].

To name just a few of the major approaches that relate to this research, it is possible to mention works that rely on changes in behavioral patterns [135,136], comparisons with non-virtual exposure treatments [137], transfer of the sense of presence and attribution to meaning due to the medium [138] (which has the strongest relation to the current research suggestions), different levels of immersion and corresponding stages of exposure meaning [139], building of a library of scenarios with proposed targeted emotions [140], storytelling as therapeutic self-expression and identity affirmation treatment [141], trauma-focused therapy (close to the current approach as we look upon trauma as a break in the narrative) [142], and mindfulness to increase the sense of presence and control on the situation and the self [143]. Modification of VR exposure due to biofeedback data is also an important feature of such therapies that may be relevant to the current research proposal as well as the opportunity to realize exposure environments that would not be possible to experience in vivo [54,144].

Prominent research work on the treatment of military PTSD desires that researchers “increase the diversity of the VR scenario content and improve the customizability of stimulus delivery to better address the needs of patients who have had a diverse range of trauma experiences” [145]. Another study focusing on combat-related PTSD emphasizes the need for emotional processing in the design of the immersion scenarios because memories are connected to meanings, and in this case, with beliefs such as “the world is a dangerous place” [71,146]. This study proposes mechanisms for symptom reduction that involve activation and emotional processing, anxiety habituation, cognitive reprocessing of traumatic meanings, learning of new responses to previously traumatic stimuli, and an integration of new information into the traumatized structure of the self, which can be related to a sequence of therapeutic processes that will be proposed here. Moreover, as Riva et al. [115] suggest, VR now has the ability to alter the experience of the body and facilitate cognitive changes by designing targeted environments able to simulate both the external world and the self. Effectively visualizing the internal processes that color a traumatic event is another feature of VR that makes it an active exposure technique in comparison to in vivo or imaginal exposures [63].

Research on the processes that happen during PTSD [147], particularly the way identity definition inhibits recovery [148], is an important prerequisite to any work that involves treating trauma through the recovery of the self-narrative. A very similar conceptual work offers therapeutic games with the purpose of creating virtual narratives with multiple choices, creating an opportunity for an enhanced experience of cathartic storytelling [149]. Even though not VR-based, an e-therapy for anxiety and depression shows the potential that various world experiences might have as part of CBT [150]. It offers different levels of therapeutic results achieved through psychoeducation, cognitive restructuring, problem solving activities, and skill acquisition and relaxation equivalent to different virtual “places” and the corresponding experiences (e.g., a volcanic province might be symbolic for dealing with strong emotions).

An interesting research design for overcoming war trauma through narrative suggests the following steps of the process: perceiving the environment as a construct of one’s own life line and self-image; active creation of a desired world, life, and self; agency and control over an environment through discovering objectively present environmental features; achieving agency through the discovery of possibilities for action rather than barriers; and making the cognitive system more flexible through a change in style of thinking and openness to new meanings [38,151]. The main points of this process relate very well to the implications of the current research, namely acquiring agency and control over one’s own life story by obtaining narrative meaning and therefore recovering the self. As the author suggests, “online world processing gives a beginning to choices regarding the reality in which an object wants to develop or relocate his/her identity” ([151], p. 37), which is the basis of this research proposal, namely, that one can recreate their world and identity in the virtual space and transfer it to the real world [15]. In support of the narrative account, an extensive study also claims that treating PTSD does not mean healing it, as healing can only be achieved through the essence of the storytelling [152]. The power of personal narrative as a healing method is most strongly argued for in the most severe types of PTSD related to military and sexual trauma [153].

An even more storytelling-focused approach [147] discusses the role of storytelling as a coping strategy in the aftermath of trauma. In relation to previous research about the break in the narrative of the self [33,72], here, traumatic changes are seen as a cause for the interruption of one’s story and even disconnection of the self-dialogue as self-identity is narratively constructed. In this sense, therapy is a sense-making process. According to this study, which discusses complex PTSD as an example of having a dissociative effect on the self, narratives classify events and perspectives on the self and so can work on changing maladaptive behaviors and thought distortions to create a solution-focused and action-oriented treatment experience. In this sense, the narration of traumatic memories is a highly effective intervention aided by the role of storytelling, and most importantly, self-narrative is described as a process of valuation in which the self rearranges meaning to achieve self-organization. Then, if storytelling is a coping mechanism, the author restores the self from trauma during a narrative experience and through empowerment of innovative versions of that self.

It seems that increasingly relevant storytelling-related studies are being produced in recent years and such strategies for addressing trauma might prove promising in the overall quest for health and self-actualization in the case of adverse life events [154,155]. All these therapeutic approaches show that attributing meaning to adverse events can help the self in reestablishing its unity and agency while adapting the VR scenario to each specific case, as this paper also proposes. Its concept, though, relies on the philosophical idea of the constitution of the identity of a healthy self, which connects with the storytelling nature of the human mind. What exactly does this account suggest?

### 5.2. The Narrator and the Protagonist

Although philosophically speaking a narrative can be perceived as a fiction [19], it can be modified and serve as a cognitive restructuring story, which when internalized, would help the person immersed in VR to create a new narrative self in a new story. If the self is perceived as an “abstractum” [19], then the author of the narrative should not be completely associated with the protagonist. This concept can be utilized in actual cases for therapeutic recovery of the identity [156]. This concept indicates that even though selves are constructions for keeping track of the history of the body, they are still considered to be constructs and can be addressed in a VR with positive results. 

For example, such a stance toward one’s life story could help a person suffering from trauma step out of the traumatic story and not identify with their image (protagonist) of a broken self, with the purpose of recreating the story of the past into a more meaningful story (as a narrator) or creating a new narrative for the protagonist to keep track of their life story in a new paradigm (as suggested in Figure 2) [157].

### 5.3. Separation from the Self as A Therapeutic Effect

Even if complete separation of the narrator from the protagonist is not possible, it is possible to utilize the mirror association explained in Section 4 and to use stepping out to internalize the fact that the person looking at his or her reflection is the author of the narrative and the origin of the reflection. Such realization could result in an understanding and acceptance that a person can make changes to this image, similar to how a person can change their image when using a mirror. 

This indicates that the acknowledgement of one’s ability to be author or narrator of a story can be achieved through self-reflection such as that achieved with mirrors. To some extent, such self-reflection can be achieved through exposure in VR when identifying with a virtual self-representation (an avatar, for example) or through observing and analyzing one’s own reactions to various stimuli in VR [16,106]. 

For example, some people cannot realize that they are more sensitive than other people to certain stimuli, which might make them more prone to developing PTSD. However, exposure in VR could help achieve such a realization and serve as a therapeutic effect for cognitive restructuring to help the person recreate their narrative and their self-concept as proposed here. In such cases, careful ethical consideration of the individual’s characteristics will be necessary because one type of trauma might affect certain people more deeply than expected due to their own personal story context, which should also be assessed with care.

### 5.4. Is the Concept of a Self Necessary?

From a philosophical point of view, the question of whether or not there is a self might influence the effectiveness of exposure in VR, because a person’s associations with real life events will be more or less flexible (for example, a person can escape from thinking patterns such as “I cannot change myself” if the person realizes there is no “true” self, only an idea of a “self”). 

**Figure 3:**
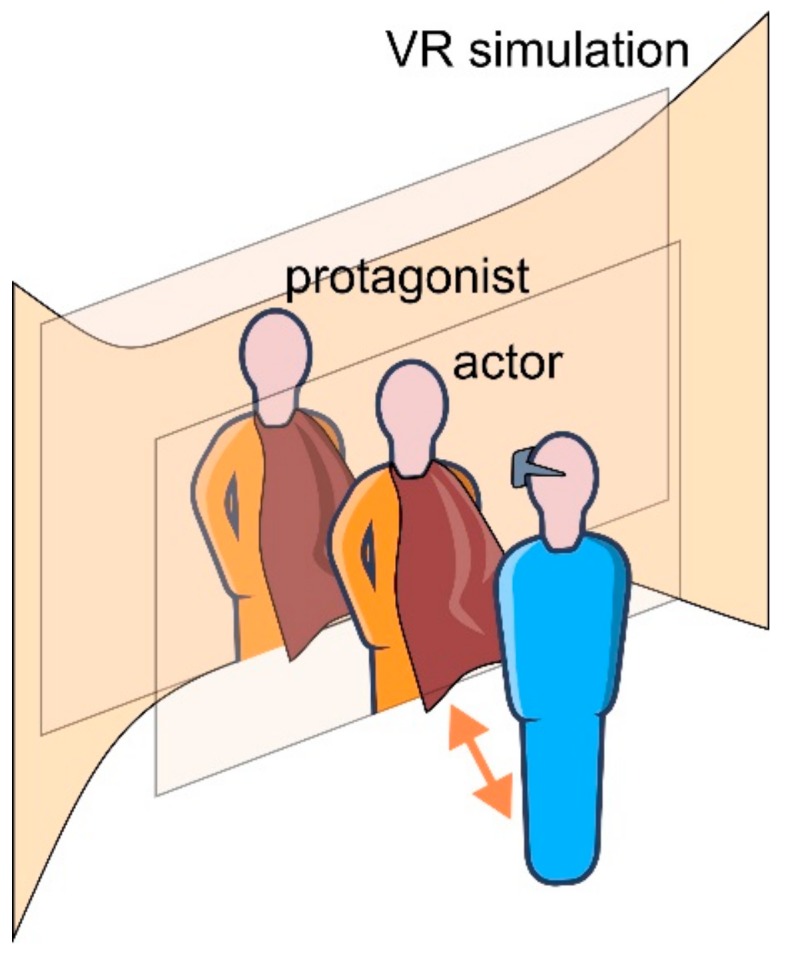
Actor, protagonist, and author in Virtual Reality. In continuation of the therapeutic effect described in Figure 2, after the achieved cognitive restructuring, here, the person (the author/the narrator) exposed to the Virtual Reality simulation might go further on their own and achieve greater transformation through separation from the roles they have about his or her self and through realization that he or she is the author of the story about their life. This can happen by seeing himself or herself as an actor who is playing the role of the protagonist in the whole story. With this, the narrative therapy in Virtual Reality can lead to an increased sense of ownership and agency to recover the self.

Accepting that there is a self that a person creates and that this self is an actor in the narrative is a distinct perspective that could make VR experiences more efficient therapy tools. An additional step would be to accept that there is such a self as an actor and that there is a person that stays behind that self as an author of the narrative. This person’s self can be viewed as its author through the acts of creating or retelling the narrative and through the changes in the protagonist or the actor that this author associates with (Figure 3). Distinguishing between these views might result in further fruitful treatment ideas and should be the subject of further studies.

## 6. Resolutions and Results from Authoring the Story

To further speculate what happens during VR exposure and to support the idea of the creation of a new story of the self, it seems plausible that reconstructing and internalizing a new story in VR is an effective and fast way to recover from psychological trauma as these experiences add to the human ability for storytelling, while offering a medium that is flexible enough to alter the storyline according to the needs of the patient as the treatment advances [38]. 

According to the concept of the narrative of the self, people can create “selves” and their stories in a virtual environment as they “weave different subplots into a single narrative” [16]. Therefore, it is plausible to recreate these selves or these characters in a storytelling and story-making environment in VR. Viewing the story as linear and occurring in VR helps a person detach from negative viewpoints acquired from real life experiences and see this viewpoint as a simulation or a story that one can review. Seeing their experiences within a story construction process could help a person gain control of the situation, as the story would resemble the situation from real life, but would also offer differing direction and choice when presented in VR.

As a person’s thoughts become narratives that become beliefs that shape reality, one can achieve realization of the authorship of their own life story and regain control of his or her self if it is suffering from a traumatic breakdown. However, the ways in which trauma is defined, discussed, and associated with self-identity can have immense moral implications and should be carefully considered [158].

### 6.1. Seeing Life as a Storyline

The narrative concept can further explain the idea of seeing one’s life story as an editable narrative aiding the success of VR exposure. According to this idea, we understand our lives as narrative in form and live according to this construct [20], which helps us experience and interpret our present not as isolated moments, but as part of an ongoing story. This means that having a narrative and being its author helps people understand and constitute themselves in the process experienced as “life”. This process has the form of a storyline that can change its direction due to events out of our control [38].

Reliving the challenges in a different way while trying to elevate the emotions connected with them to a higher level (e.g., from regret to motivation) might help a person experience catharsis and enrich them, rather than rob them of something essential in life. Turning back toward the event and focusing on the perspective gained might help a person escape a negative fixed mindset, re-contextualize the past, and overcome the victim attitude to create opportunity and inspiration to move on.

Being put into the story and re-experiencing it in VR while retelling it, might change its context and help a person embody a different view of reality [159,160,161].

### 6.2. Background of The Story

In addition, a certain background exists in any narrative. For example, “[t]he experience of winning the lottery will, for instance, be a different experience for someone immensely wealthy, someone who has lived a life of crushing poverty, and someone who has struggled unsuccessfully with a gambling addiction” [21]. A narrative’s background is very important to consider when designing various VR programs. In particular, as is shown with cases of PTSD, under the same definition of traumatic stress are completely different triggering events in which one’s actions or lack of action, choices or lack of choice, created the traumatic experience. Therefore, the feeling of guilt, the direction of anger toward others or one’s self, the blame or the moral conflict are placed in completely different emotionally charged stories. How one would assess these stories, find, and defend their ethical bases and help one overcome the complex feelings associated with interpretations of the stories is specific for each case.

This concept can support the hypothesis that narrative explanations are not necessarily valid for one’s whole life. One can create different narratives while being immersed in a VR story. People employ storytelling logic to describe, explain, and choose their own behavior [20], especially when necessary such as when facing difficult situations. It appears that this process can happen in VR, where people can design and create their own stories. This means that when new explanations are necessary, people can rewrite the story if it is experienced as a game [162] or, as discussed in Section 3, as part of one’s journey in life. Therefore, adding explanations to past events, participating in the design of the VR story and being an active protagonist as well as the author of a story could have immense therapeutic effects on the self in the quest to overcome trauma.

### 6.3. Explanation and Meaning of The Story

Using VR to explain that one’s single storyline (image of life) has ended when some traumatic event occurred could enable this person to understand that they have the chance to explain and retell the event through gradual and repeated exposures, making it fit their own overall life story in a new way. This is the essence of story recreation in VR. In other words, the traumatic event can finally make sense when explained within a new narrative framework that makes sense to the person who experienced the trauma [38]. 

Schechtman suggests that “the limits of a person are determined by the limits of a narrative, and the integrity of a single person consists in the unity of a narrative” [16]. When one’s life story expands in a different direction, even if this direction might seem undesirable at first, new insights on the self and new opportunities to grow as a person occur. Perceiving such possibilities as a healthy narrative and experiencing them in VR could remind the person of their narrative capability and help them recover by obtaining control over their life story.

## 7. Proposal for Design of VR Stories with a Narrative Concept

Using the self-narrative and VR-conceptual framework discussed in the previous sections, this theoretical research proposes a gradual process to achieve a therapeutic effect through experiencing events related to trauma, designed as follows:

- Interviewing the patient to create a detailed personalized story;
- Designing exposure and reliving/retelling the traumatic event with the patient’s active participation;
- Changing the point of view and gaining a separate perspective of the traumatic events and the surrounding context, in addition to the existing background of the personal narrative;
- Processing the events utilizing cognitive restructuring of unhealthy narratives and notions about one’s self;
- Re- or overwriting of events that are difficult to process with fewer negative or more meaningful ones;
- Including/integrating the story into the overall life narrative;
- Resolving the conflicts in the story;
- Adding explanation/meaning/closure within the classical principles of storytelling as necessary for the specific case;
- Therapeutic directing of authoring and owning the recovered or newly created story; and
- Achieving recreation of a healthier self.

The retrospective storytelling and addition of meaning can aid the process of gaining perspective and making sense to help one’s self feel whole again [163]. Such a flexible design should consider the many ethical issues related to what kind of story is acceptable and in what way the person can obtain recovery. In addition, AI could help in the design of the storytelling environment, as the environment should be very adaptable to the personal scenarios of each individual case. The rationale behind using different narrative experiences after the re-experience of the traumatic events and resolution of underlying negative concepts can bring about the storytelling nature of the human self in search of explanation and growth, as seeing a deserving end of a fictional story can still have a healing effect and support one’s belief system and sense of authorship and ownership of their life story.

### 7.1. Proposals for Scenario Design

This paper proposes the building of therapies based on conclusions made from the conceptual framework utilizing the theory of the narrative self.

Three scenarios are presented below and show potential interventions for psychological trauma resulting from different events. They illustrate the narrative dialogue sequence, the aim, the means, and the expected outcomes of such narrative reconstruction. Moreover, some ethical limitations that may arise from such scenarios are also discussed below.

Case 1. A patient who could not save and, therefore, lost a close relative during a tsunami who developed PTSD because of the traumatic experience (Table 1).

Case 2. A patient attacked on the street, robbed, and heavily beaten with a serious traumatic response from the experience (Table 2).

Case 3. A patient who was seriously injured in a car crash, which killed the other driver, and experiences severe PTSD (Table 3).

The therapeutic setting of the three scenarios suggests completing levels 0 and 1 before experiencing level 2. However, the therapist should only advance the patient to level 2 if they consider them ready, capable, and recovered. The design of the experiences in level 2 may vary; its main idea, though, is to present a cathartic and enriching experience that will resolve psychological tension and present a forward-looking conclusion to a negative story [164,165,166].

### 7.2. General Limitations and Challenges

The paper is a theoretical discussion of the hypothetical use of VR in therapy. It considers how highly personalized and customizable VR scenarios and narratives can be created and edited by the participant/protagonist on the spot, so that they can reimagine their life story in a meaningful way that is therapeutic rather than traumatic to recall. However, with currently available technology, detailed editing of VR scenarios is still very limited. Therefore, such a design would require advanced technical elements and a very resource-intensive user interface to assemble into a coherent narrative. This is currently difficult to achieve. In the near future, when greater resources and new technologies become available, such applications of VR will become possible and assist in bringing about significant advances in therapy and general healthcare.

In detail, the limitations of VR for therapeutic purposes can be technological and conceptual [167]. More concrete limitations pertain to the ethical aspects of the usage of VR for the treatment of serious health conditions. First, the usage of a tool to simulate alternative realities and scenarios that might seem more appealing than real life outcomes are more or less dangerous with the proposition that they might provoke the desire to escape from reality entirely and form an addiction to the experience as a substitute for facing real challenges. In this sense, it is necessary to have such exposure techniques carefully supervised by clinicians and therapists aware of these challenges and equipped with methods to avoid unfavorable results.

In connection to this, the exposure might prove to be too intense for the patient, so a careful and gradual administration of the experience specifically designed for the patient is an important consideration relating to this proposal.

However, there are also considerations regarding the severity of trauma that may backfire in cases of serious physical disability resulting from injury that have led to changes in lifestyle when it is difficult to imagine the same life as the one before the traumatic event. Still, even such cases can lead to recovery, growth, and search for meaning [168,169], but require an understanding of how very prolonged and serious trauma affects identity and results in major changes in lifestyle or personality as this study connects with the philosophical account of the narrative self-identity. For example, necessary factors to consider are significant changes in lifestyle such as relocation and physical damage resulting in irreversible psychological traumatic conditions. Moreover, internal conflicts that are not resolved or might be unresolvable due to factors such as missing persons, etc., may also backfire as well as controversial thoughts and beliefs that are too harsh or not at all subject to change or influence.

### 7.3. Challenges Specific to VR

In addition, distinguishing between when VR is appropriate and when it is not applicable for clinical treatment cases is an important role to be addressed before it is sophisticated enough to use in practice [170]. The quality of the exposure and the cost of its design as well as the technical literacy and the ability to operate the hardware are other aspects of the VR-specific problems to be addressed before employing such methods of treatment [171].

As discussed, addictive behavior is a potential risk for users of VR technology [172,173]. In addition, prolonged experience in a VR simulation can provoke a condition known as cybersickness [174]. Accordingly, it is possible to imagine that if VR creates an intriguing and highly immersive story, then participants can face more challenges when it becomes necessary to separate what is “real” from what is “unreal”, compared to the narrative creation in non-virtual worlds as in imaginal exposure for treatment of traumatic disorders. These and other limitations related to therapy as well as the methodological limitations should be discussed and addressed to determine the suitability of VR for treatment in the context of the restoration of the self-narrative and the kind of dangers it can present in the context of the fast-paced digitalized lifestyle. For example, moral questions regarding the usage of robot soldiers, fears of AI domination over the human race, and the possibility of a Matrix-like lifestyle can show how a mismatch between the initial idea for utilization of technology and the potential reality could happen.

To support the effectiveness of therapy in VR, researchers should consider what dangers there are in connection with the utilization of VR, especially when handling healthcare problems. The emphasis on soldier treatment in VR is creating an attractive image of VR in society without fully acknowledging whether this treatment method could fit all types of patients suffering from PTSD. For example, VR could be used effectively for the treatment of the fear of heights as it provides sufficient safety to perform the treatment in comparison to other possible exposure methods. However, it should be considered that the exposure could cause the patient to become too well adjusted, creating potentially harmful fearlessness. This concern especially applies to soldiers who become too immersed in their training and start to see the reality of war as some form of game [131]. More research is necessary to define which health conditions are best treated in VR. As the focus here is the self-narrative, the subject of analysis will be PTSD as a disorder that affects one’s story in a very thorough way.

Cybersickness has been explored in studies investigating the possible applications ahead of VR as well as research on the limitations of VR itself as in studies of the sensation of presence and cybersickness in applications for rehabilitation [175]. According to this research, these two phenomena—presence and cybersickness—seem to be closely related. The influence of visual and vestibular stimuli on cardiovascular responses was studied to present how VR technology expands sensory effects as well as physical activities. The authors focused on developing regular exercise practices in VR that will bring about necessary therapeutic effects without the negative influences, which should not be neglected when utilizing VR.

VR simulations induce sensations through multi-sensory stimuli; however, these can be “inappropriate to each other or slightly different from those experienced in the real world” [175] and “could evoke symptoms of cybersickness, even though such stimuli would excite the users and increase their sensed feeling of reality” [175]. It seems that the experience in the virtual environment can be “stimulating,” in the sense of excitement, and at the same time somewhat “sickening” to the people experiencing differing events in it. This twofold effect might be the reason why VR can be utilized for therapy in the first place. The mixture of positive and negative effects resembles a real situation such as the one experienced during trauma. For example, a person in the military can feel very “alive“, active, and stimulated during combat and at the same time feel scared, furious, or disgusted from the experience. In the same way, a person surviving a disaster might feel a rush of adrenaline when trying to save his or her life, while at the same time feeling horror because of the extreme situation.

Let us return to the analysis of cybersickness. The research cited above claims that “preventing (an) unpleasant situation is a key point for sustaining sufficient effectiveness and motivation” [175]. This means that soldiers experience the VR simulation in the same way they meet the adversities of war in reality. This desire to overcome obstacles leaves the soldiers active and motivated to escape from the same sensations they are having during combat environments. Similarly, a person escaping from disaster is alert and reasonable in their actions meant for survival. In other words, the soldier wants to complete the mission, stay alive and unharmed, and end the stressful situation as soon as possible and as effectively and safely as possible; in the same way the person running from a disastrous tsunami tries their best to stay alive and unharmed, possibly help others to survive, and reach safe ground as soon as possible. Therefore, their motivation to survive can be realistically triggered in a VR simulation. However, when these people are experiencing the simulation of similar events in a VR environment, the “mismatch between the visual and vestibular systems can disturb the autonomic nervous regulation and lead to symptoms of motion sickness” [175]. This means that besides the fact that VR therapy enables many processes to happen faster in the virtual environment, these features of the virtual environment itself bring additional stressors that could be neglected, especially in the beginning of the VR experience when the new stimulating experience provokes euphoria in the patient. In this sense, the main feature of the VR therapy, namely as a useful tool for shortening the therapeutic process in an effective way, may also bring a new set of problems that are completely absent in the case of all other therapeutic practices available for PTSD (i.e., imaginal exposure).

Diving even further into the dangers VR can pose, it is possible to discuss the results a total virtual customization of needs and immediate personalized gratification might have [176]. The aftereffects of the persistent usage of VR can bring about a completely new field of research and the necessity for accompanying guidelines [177]. There are other risks that health intervention technologies might have that can be generally summarized as risks of addiction, stigmatization, isolation, bullying, or even risk of cultural issues that can pertain to the exposure in VR as well since the personal story representation should be considered very carefully with all of these factors in mind [178].

In this sense, the theoretical challenges in front of the self-narrative paradigm are also a point of further consideration and discussion, especially when there is a sense of no coherent self, due to too strong a trauma. Nevertheless, the narrative self-account in VR therapy might continue to be a helpful solution even in such extreme cases.

## 8. Final Remarks and Outlook to The Future

VR researchers [179] suggest that VR is in the stage of transition when new methods of expression need to be defined to bring about a paradigm shift of the medium, similar to the one that took place when theater transitioned to movies in popular culture. Now, several years later, we are discussing the possible ways this medium can offer deeper immersion and stronger storytelling than other media, and better therapeutic effects than other exposure techniques as a novel experience for the narrative self.

This paper adds to the existing research on narrative exposure therapy with a novel proposal for VR to be used as a medium to reconstruct one’s perception of reality and one’s own life story. The original idea presented here is based on the philosophical concept of life as a narrative that is used to explain its events and form and contain the notion of a self. The proposition that one can once again feel they are the author of their life story can empower experiences that return ownership of one’s narrative self, rebuild the meaning of one’s life story, and create a healthier reality [180]. These concepts could be applied to the practical design of immersive environments meant for larger scale results such as behavioral change, health improvement, self-development, and so forth.

We argue that the findings of this research will be useful for future designs of VR environments, especially when considering the personal output of those who use these environments while preserving their own life stories with all the ethical implications. Such a process of narrative self-recreation can be part of the utilization of VR and AI that is in-between entertainment and treatment and is adaptable to the everyday needs of each and every person interested in simulated realities and the real stories behind them. These concepts can also be used to improve life quality not only for those seeking therapeutic treatment, but also for those in the general population seeking resilience, self-actualization, and better and healthier lifestyles. 

## Figures and Tables

**Table 1:** Tsunami survivor case.

Depth of Experience	Content of Exposure in VR	Means, Aims and Expected Results
0 level (exposure)	Re-experience the event	Acknowledge the feelings of helplessness, guilt and grief
1st-level (resolution)	Meet with a virtual image of the relative in a different setting again	Explain why/how he or she could not help, ask for forgiveness, properly say goodbye
2nd-level (different narrative experience)	Meet with a virtual image of the relative in a safe fantastic world (possibly underwater)	Manage to save the relative from another dangerous situation, talk and share some positive feelings/experiences together

**Table 2:** Assault survivor case.

Depth of Experience	Content of Exposure in VR	Means, Aims and Expected Results
0 level (exposure)	Re-experience the event	Acknowledge the feelings of helplessness, fear and pain, as well as anger if present/detected
1st-level (resolution)	Meet with a virtual image of the attackers in a different setting	Explain how terrified he or she felt, find understanding and, if possible, forgive the deed
2nd-level (different narrative experience)	Meet with a virtual image of the attackers in a different and safer setting	Manage to fight back when they try to attack him or her OR manage to escape and find himself/herself in a very pleasant and relaxing state OR manage to save a different person from a similar situation

**Table 3:** Car crash survivor case.

Depth of Experience	Content of Exposure in VR	Means, Aims and Expected Results
0 level (exposure)	Re-experience the event	Acknowledge that he or she is alive and that the circumstances led to a death; he or she is not consciously committed, but feels guilt and pain regarding the event
1st-level (resolution)	Meet with a virtual image of the person from the other car (possibly while stopping at a gas station)	Explain the feelings of guilt and horror of the event and ask for forgiveness, say goodbye
2nd-level (different narrative experience)	Meet with a virtual image of the person from the other car in a non-road related environment	Get to know each other, talk about family, spend positive time together, feel like friends
